# Characterization of the complete mitochondrial genome of *Elanus caeruleus* Desfontaines, 1789 (Accipitriformes: Accipitridae)

**DOI:** 10.1080/23802359.2022.2060769

**Published:** 2022-04-04

**Authors:** Haoran Luo, Wenzhen Fang, Qingxian Lin, Xiaolin Chen, Xiaoping Zhou

**Affiliations:** Key Laboratory of the Ministry of Education for Coastal and Wetland Ecosystems, College of the Environment and Ecology, Xiamen University, Xiamen, Fujian, People’s Republic of China

**Keywords:** Black-winged kite, mitogenome, next-generation sequencing, phylogenetic analysis, Elaninae

## Abstract

The complete mitochondrial genome of the *Elanus caeruleus* was sequenced via next-generation sequencing. The circular mitogenome is 18,898 bp in length, containing 13 protein-coding genes, 22 transfer RNA genes, 2 ribosomal RNA genes, a control region and a pseudo control region. The sequences (1210 bp) in the middle part of the two control regions are complete identical to each other. The gene order of the *E. caeruleus* mitogenome is identical to those of other Accipitridae species. The phylogenetic analysis indicated that *E. caeruleus* formed a basal lineage sister to other species within the Accipitridae family.

The black-winged kite, *Elanus caeruleus* (Desfontaines, 1789), is a small diurnal bird of prey belonging to the Accipitridae family. It has a very large distributed range encompassing Africa, India, southeastern Asia and southwestern Europe (Balbontín et al. 2008). This species is listed as Least Concern (LC) on the International Union for the Conservation of Nature Red List of Threatened Species (BirdLife International 2019). However, in China, it has been classified as a second-class key protected wild animal (National Forestry and Grassland Administration 2021). So far, the complete mitochondrial genomes have been reported for several species from the other subfamilies within the Accipitridae (for example, Choi et al. 2021), but not including species from the subfamily Elaninae. In this study, we sequenced the complete mitogenome of *E. caeruleus* and analyzed its phylogenetic position within the Accipitridae.

Muscle tissues of *E. caeruleus* was collected from a dead individual in Liancheng, Longyan City, Fujian Province, China (116.75°E, 25.72°N) and preserved in 95% ethanol. The specimen was stored at −80 °C in the laboratory at College of the Environment and Ecology, Xiamen University, Xiamen, China (https://cee.xmu.edu.cn/, Xiaoping zhou, xpzhou@xmu.edu.cn) under voucher number CEE2016Aves-EC-01. Genomic DNA was extracted using the EasyPure^®^ Genomic DNA Kit (TransGen Biotech Co., Ltd., Beijing) and then sequenced on the Illumina NovaSeq 6000 sequencing platform with a pair-end sequencing protocol of 150 bp read length (PE150). FastP (Chen et al. 2018) was used to remove adaptors and low-quality reads. Clean pair-end reads were assembled into a complete mitogenome using GetOrganelle v1.7.5 (Jin et al. 2020) and total 21 different assemblies were generated. After annotated by MITOS2 (Donath et al. 2019), we found that the sequences of the 21 assemblies were different in the 3′ end of the two control regions. Thus, we amplify the of the 3′ end of the two control regions with two primer pairs (ARCR372F + ARProR and ARCR372F + AR12SR1, Zhou et al. 2014) and aligned the sequences of the PCR products with the 21 assemblies to identify the correct result.

The circular mitogenome of *E. caeruleus* is 18,898 bp in length (GenBank accession OK662584), including 13 protein-coding genes (PCGs), two ribosomal RNA genes (12S rRNA and 16S rRNA), 22 tRNA genes (tRNAs), a control region (CR) and a pseudo control region (ΨCR). The overall A + T content is 53.7% (29.0% A, 24.7% T, 14.3% G, and 32.0% C).

All the PCGs use ATG as start codon except for *ND3* using ATC and *ND5* using ATA. The most common stop codon is TAA, while *ND1*, *COX1* and *ND6* end with AGG, *ND2* ends with TAG, *COX3* and *ND4* end with a single T. Additionally, *ND3* has one extra cytosine in the 174th nucleotide position, which is presumed not to be translated (Mindell et al. 1998).

The gene order of the *E. caeruleus* mitogenome is identical to those of other Accipitridae species. The CR (1795 bp) is located between *tRNA^Thr^* and *tRNA^Pro^*, whereas the ΨCR (1586 bp) is located between *tRNA^Glu^* and *tRNA^Phe^*. The sequences (1,210 bp) in the middle part of the CR and ΨCR are complete identical to each other, which contain several conserved boxes, such as F, E, D, C and bird similarity box, generally existing in the Avian mitogenome control regions (Zhou et al. 2014; Choi et al. 2021).

The concatenated nucleotide sequences of the 13 PCGs of the *C. caeruleus* mitogenome and 14 published mitogenomes from differ genus (one species per genera) within the Accipitridae were used for phylogenetic analysis, and *Sagittarius serpentarius* from Sagittariidae and *Pandion haliaetus* from Pandionidae were used as the outgroups. A Maximum-likelihood (ML) tree with 1000 bootstraps was constructed by IQ-TREE v1.6.2 (Trifinopoulos et al. 2016) using the substitution model GTR + F + I + G4, which was selected by Modelfinder (Kalyaanamoorthy et al. 2017). The result (Figure 1) indicated that *E. caeruleus* formed a basal lineage sister to other species within the Accipitridae family, which was in consistent with previous molecular studies (Starikov and Wink 2020).

**Figure 1.**
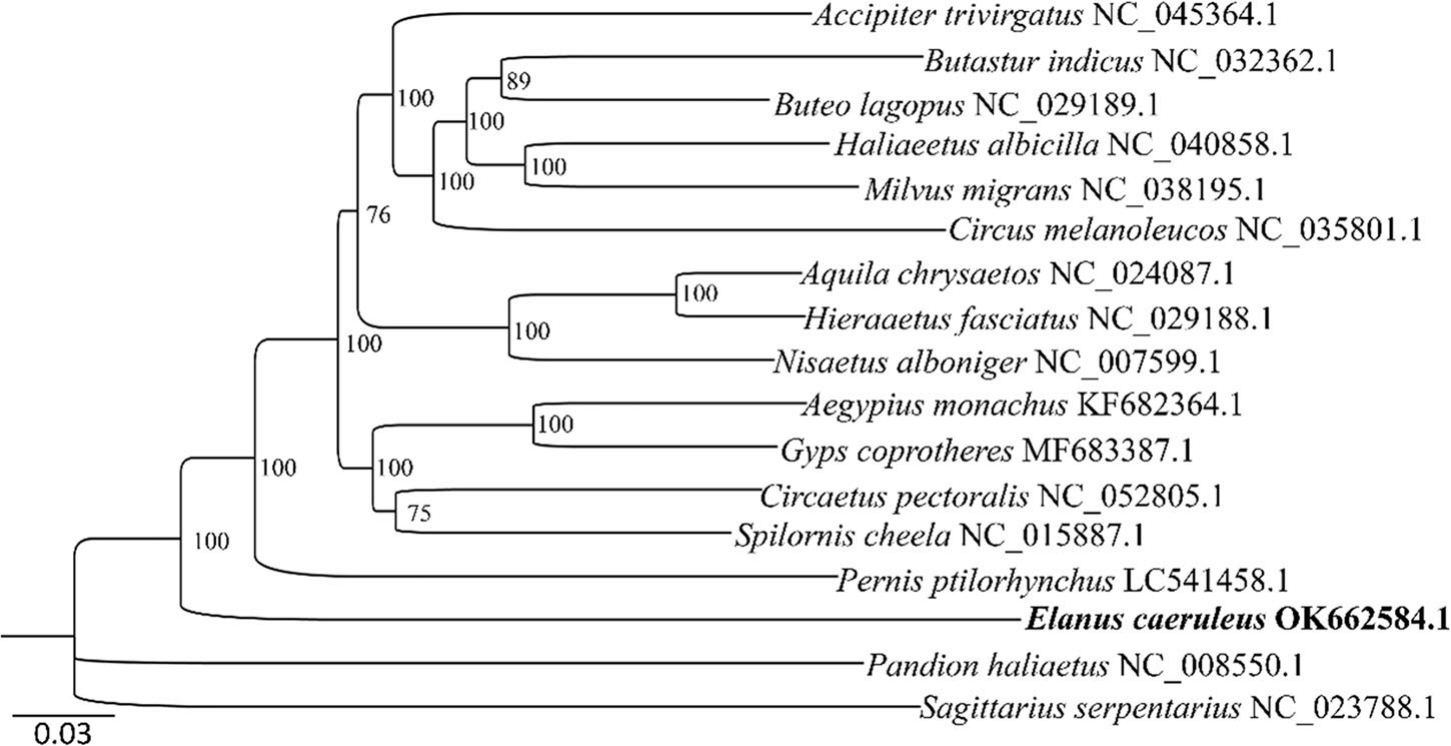
The maximum-likelihood (ML) tree of *E. caeruleus* and other 14 species within Accipitridae based on the concatenated nucleotide sequences of 13 mitochondrial PCGs. *S. serpentarius* and *P. haliaetus* were used as the outgroups.

## Data Availability

The mitochondrial genome sequence in this study is openly available at GenBank (https://www.ncbi.nlm.nih.gov/) under the accession number OK662584. The associated BioProject, SRA, and Bio-Sample numbers are PRJNA775080, SAMN22602020, and SRR16888995, respectively.

## References

[CIT0001] Balbontín J, Negro JJ, Sarasola JH, Ferrero JJ, Rivera D. 2008. Land-use changes may explain the recent range expansion of the black-shouldered Kite *Elanus caeruleus* in southern Europe. IBIS. 150(4):707–716.

[CIT0002] BirdLife International. 2019. *Elanus caeruleus*. The IUCN Red List of Threatened Species 2019: e.T22695028A152521997. [accessed 2021 Nov 01] 10.2305/IUCN.UK.2019-3.RLTS.T22695028A152521997.en.

[CIT0003] Chen S, Zhou Y, Chen Y, Gu J. 2018. Fastp: an ultra-fast all-in-one FASTQ preprocessor. Bioinformatics. 34(17):i884–i890. 30423086 10.1093/bioinformatics/bty560 PMC6129281

[CIT0004] Choi EH, Enkhtsetseg G, Baek SY, Hwang J, Park B, Jang KH, Ryu SH, Hwang UW. 2021. Complete mitochondrial genome of a hen harrier *Circus cyaneus* (Accipitriformes: Accipitridae) from South Korea. Mitochondrial DNA B Resour. 6(1):185–186. 33537437 10.1080/23802359.2020.1860700 PMC7832496

[CIT0005] Donath A, Jühling F, Al-Arab M, Bernhart SH, Reinhardt F, Stadler PF, Middendorf M, Bernt M. 2019. Improved annotation of protein-coding genes boundaries in metazoan mitochondrial genomes. Nucleic Acids Res. 47(20):10543–10552. 31584075 10.1093/nar/gkz833 PMC6847864

[CIT0006] Jin JJ, Yu WB, Yang JB, Song Y, dePamphilis CW, Yi TS, Li DZ. 2020. GetOrganelle: a fast and versatile toolkit for accurate de novo assembly of organelle genomes. Genome Biol. 21(1):241. 32912315 10.1186/s13059-020-02154-5 PMC7488116

[CIT0007] Kalyaanamoorthy S, Minh BQ, Wong TKF, von Haeseler A, Jermiin LS. 2017. ModelFinder: fast model selection for accurate phylogenetic estimates. Nat Methods. 14(6):587–589. 28481363 10.1038/nmeth.4285 PMC5453245

[CIT0008] Mindell DP, Sorenson MD, Dimcheff DE. 1998. An extra nucleotide is not translated in mitochondrial ND3 of some birds and turtles. Mol Biol Evol. 15(11):1568–1571. 12572620 10.1093/oxfordjournals.molbev.a025884

[CIT0009] National Forestry and Grassland Administration. 2021. Announcement of the ministry of agriculture and rural affairs of the national forestry and grassland administration (No. 3 of 2021) (List of National Key Protected Wild Animals). http://www.forestry.gov.cn/.

[CIT0010] Starikov IJ, Wink M. 2020. Old and cosmopolite: molecular phylogeny of tropical-subtropical kites (Aves: Elaninae) with taxonomic implications. Diversity. 12(9):327.

[CIT0011] Trifinopoulos J, Nguyen LT, von Haeseler A, Minh BQ. 2016. W-IQ-TREE: a fast online phylogenetic tool for maximum likelihood analysis. Nucleic Acids Res. 44(W1):W232–235. 27084950 10.1093/nar/gkw256 PMC4987875

[CIT0012] Zhou X, Lin Q, Fang W, Chen X. 2014. The complete mitochondrial genomes of sixteen ardeid birds revealing the evolutionary process of the gene rearrangements. BMC Genomics. 15(1):573. 25001581 10.1186/1471-2164-15-573 PMC4111848

